# *EHP’s* Policy on Integrity of Published Research

**DOI:** 10.1289/ehp.13245

**Published:** 2009-09

**Authors:** Hugh A. Tilson, Jane C. Schroeder

**Affiliations:** Editor-in-Chief, *EHP* E-mail: tilsonha@niehs.nih.gov; Science Editor, *EHP*schroederjc@niehs.nih.gov

Fifteen years ago, *Environmental Health Perspectives* (*EHP)* stated in its Instructions to Authors that scientific integrity would be considered as part of the review process. Since that time, *EHP*’s policy has gradually evolved, especially with regard to competing financial interests.

*EHP*, like many other journals, is concerned that groups or individuals providing financial support might exercise controlling authority regarding the design, conduct, interpretation, or publication of research. Such controlling authority could have a corrosive effect on the independence of the individual scientist to conduct research in accordance with the principles of the scientific method. Biased research could lead to a loss of support for science by the public and those who depend on it to make informed decisions.

In 2004, Dr. Thomas Goehl, the Editor-in-Chief of *EHP* at the time, wrote an editorial (Goehl 2004) outlining several key principles concerning how the journal viewed the issue of scientific integrity:

- Contributors to the journal should be aware that the potential for competing financial interests could be present regardless of whether an actual conflict exists. Authors should be acutely aware of how relationships could be perceived by others to affect the author’s scientific judgment, especially those having differing points of view.
- Issues related to potential competing financial interests could be dealt with at some level by asking authors to provide full disclosure of potential conflicting relationships. This principle allows for the information to be available to everyone so that the reader can make his or her own judgment about the relationship.
- Disclosing potential competing financial interests does not automatically diminish the credibility of the research. Failure to disclose a competing interest, however, could jeopardize the credibility of authors if an actual conflict were discovered at a later time.

In his editorial Goehl (2004) clearly articulated the journal’s view with regard to competing interests; that is, authors should make full disclosure of potential and actual competing financial interests. Furthermore, Goehl announced that the journal would impose a 3-year ban on publication for authors who willfully failed to disclose a competing financial interest. *EHP* is one of the few environmental or biomedical journals having a stated enforcement policy. The current policy is available in *EHP*’s Instructions to Authors (available at http://www.ephonline.org/docs/admin/ita.html). We will evaluate cases of alleged violations of journal policy on a case-by-case basis.

The key principles articulated by Goehl (2004) concerning potential competing financial interests are still in effect today. When authors submit a paper to *EHP,* they must complete a Declaration of Potential Conflicts of Interest form (available online at http://ehp.niehs.nih.gov/cfi.pdf). If an actual or potential conflict exists, then authors are expected to place a check-mark in the appropriate blank on the form and briefly describe the relationship. What is important is that full disclosure is made at the time the manuscript is submitted. Authors are also asked to make a general statement about the actual or potential competing financial relationships in the Acknowledgments section of their paper. *EHP* staff and the Ethics Coordinator evaluate the Declaration of Potential Conflicts of Interest form and manuscript before the paper is sent for peer review. Details disclosed on the Competing Financial Interests Declaration form are treated as confidential information.

Authors sometimes have difficulty discerning if a specific relationship could be considered as a potential competing financial interest. The Competing Financial Interests Declaration form and Instructions to Authors provide a number of examples of potential conflicts. These lists, however, are not meant to be exhaustive. Authors should also carefully examine the wording of funding documents such as grants and contracts to determine whether there might be an actual or potential competing interest. Potential competing interests in these documents should also be declared on the Competing Financial Interests Declaration form and in the Acknowledgments section of the paper.

We should also note that *EHP* is sometimes confronted with issues regarding potential research misconduct, such as plagiarism or data fabrication. Authors should be aware that *EHP* routinely evaluates each manuscript for possible plagiarism. Instances of documented plagiarism and allegations of data fabrication will be brought to the attention of the authors’ host institutions. Documented cases of plagiarism or data fabrication could lead to a 3-year ban on future publication by the authors or retraction of the paper.

Journals must make every effort to ensure that the integrity of published research is not tainted. If policy makers and the public were to lose confidence in the scientific process, regaining it would be a formidable challenge.

## Figures and Tables

**Figure:**
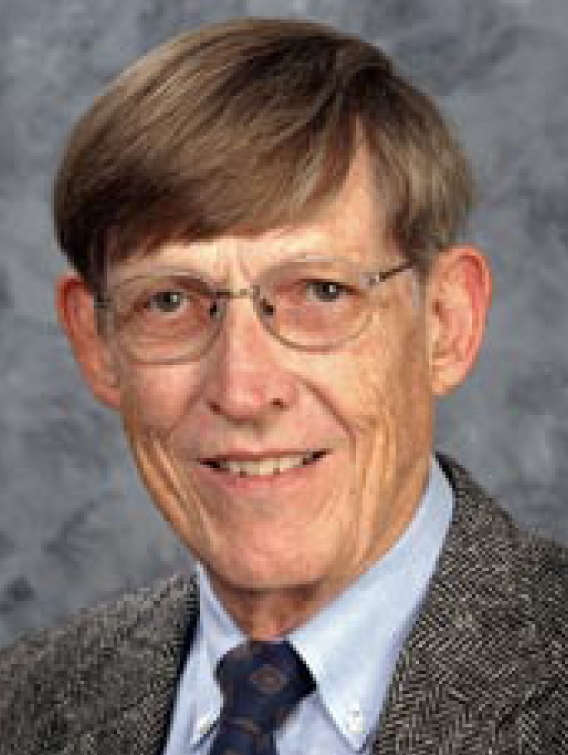
Hugh A. Tilson

**Figure:**
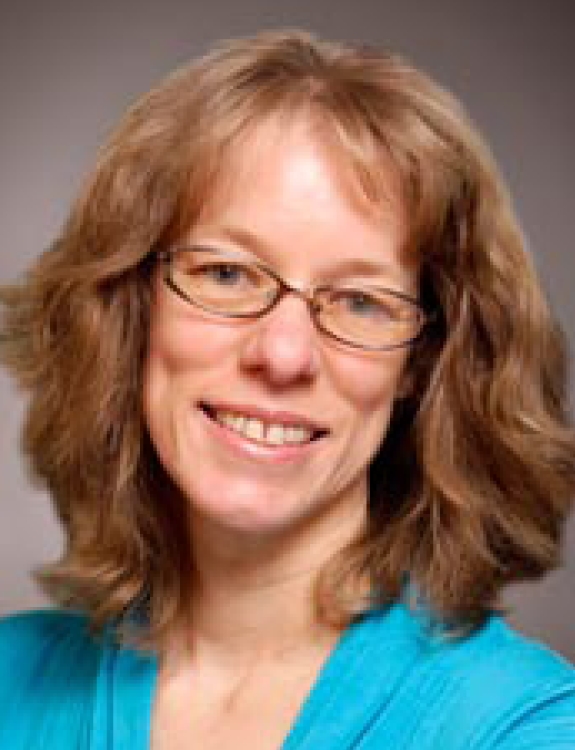
Jane C. Schroeder
